# Use of Lasers for Becker’s Nevus and Keratosis Pilaris

**Published:** 2008

Note: This section lists opinions by different doctors on specific problems. These opinions are their personal opinions and not necessarily supported by any data. The section only serves to offer options/opinions in different difficult to treat conditions.

1. Question asked: Can Becker’s nevus be effectively treated? Is it worth treating at all?
  **Responses:**
  - Beckers nevus has hairs that should be treated first with a long pulsed Nd-YAG laser followed by (a) 1 pass of 532 nm 1-2 J/s 4 mm spot size, which reduces the epidermal pigment that is pronounced in Beckers nevus followed by (b) 1064 nm 4 mm spot size 10-13 J/s till bleeding points for good results. We have tried this in our few cases.
    **Dr. SG Parasramani, Lilavati Hospital, Mumbai**
  - The best approach to a Becker’s nevus is to use a hair reduction laser along with either a Q-switched laser to lighten the lesion or a fractionated ablative laser. I have tried the Q-switched laser alone but the response was less than satisfactory. In my view, its not worth attempting. If a patient seeks treatment, we proceed only after a test patch.
    **Dr. Sanjeev Aurangabadkar, Skin and Laser Clinic, Hyderabad, India. E-mail: sanjeev.aurangabadkar@gmail.com**
  - Non-hairy Beckers nevi pigmentation can be improved by an ER:YAG laser but hairy Beckers nevi is a challenge. Here, the hairs are very resistant to therapy by either diode or Nd:YAG laser. Somehow, the response of Beckers nevi in males is less effective compared with females.
    **Dr. Omprakash, National Skin & Hair Centre, Bangalore. E-mail: drhmomprakash@yahoo.com**
  - We have treated more than 15 cases of Becker’s nevus on Harmony from Alma lasers. We have tried different options for pigment reduction, including Q-switched 1064–532 pixel, Er–YAG, LEO 540, 570, 650. Most consistent results obtained were with LEO 540 Harmony.
    **Dr. Dhepe, Consultant Dermatologist, Skin City, Pune, India. E-mail: niteendhepe@yahoo.com**
2. Question asked: Can keratosis pilaris be treated by laser?
  **Responses:**
  - To the best of my best knowledge, nothing can be done on a long-term basis for keratosis pilaris. I tried many things (lasers, chemical peelings) but improvement did not last longer than 2 months with any modality.
    **Dr. Maurice Adatto, Switzerland. E-mail: madatto@skinpulse.ch**
  - Laser hair removal cannot work in keratosis pilaris as the hairs are too small and contain little melanin. Other forms of laser also do not work consistently or produce lasting results.
    **Dr. Moshe Lapidoth, Israel. E-mail: alapidot@netvision.net.il**

